# Spontaneous Regression of Uterine Arteriovenous Malformations with Conservative Management

**DOI:** 10.1155/2017/6437670

**Published:** 2017-02-16

**Authors:** Keiko Mekaru, Sugiko Oishi, Kozue Akamine, Chiaki Heshiki, Yoichi Aoki

**Affiliations:** Department of Obstetrics and Gynecology, Graduate School of Medical Science, University of the Ryukyus, 207 Uehara, Nishihara, Okinawa 903-0215, Japan

## Abstract

Uterine arteriovenous malformation (AVM) can cause massive hemorrhage and is often treated with uterine artery embolization (UAE), which may lead to ovarian insufficiency. Thus, avoiding UAE should be considered, particularly in women undergoing fertility treatments. We present three women diagnosed with postmiscarriage AVM on color Doppler by transvaginal ultrasound imaging. They had no genital bleeding and a small mass, measuring 16–22 mm. If estradiol was >300 pg/mL when AVM was diagnosed, then a gonadotropin-releasing hormone agonist was administered. All three women underwent follow-up observation, revealing spontaneous mass disappearance. To avoid ovarian insufficiency risk with UAE, conservative management and close follow-up observation should be considered in patients with AVM without bleeding, particularly during the fertility treatment.

## 1. Introduction

Uterine arteriovenous malformation (AVM) is a rare condition, and although its precise incidence is unknown, most cases of AVM are undiagnosed. Thus, this condition may have a higher incidence than expected [1]. AVM can be congenital or acquired. Acquired AVM is believed to be caused by spontaneous abortion [2], dilation and curettage (D&C) [2, 3], uterine trauma [4], and gestational trophoblastic disease [5]. The reported incidence of AVM has been increasing [6–8]. Although emergency uterine artery embolization (UAE) can be performed to stop the bleeding, some women may require hysterectomy [3, 6, 8]. The effect of UAE on fertility and pregnancy is unclear; therefore, performing UAE for women who wish to conceive remains controversial [9, 10].

Alternatively, some reports have described the spontaneous disappearance of AVM by expectant management [1, 3, 11–15]. Therefore, considering the risks for ovarian insufficiency and placental abnormalities during subsequent pregnancies associated with UAE, we examined whether UAE can be avoided in patients with AVM, particularly in those undergoing fertility treatment. We retrospectively examined patients with AVM who were treated at our department and determined the feasibility of conservative management as a treatment option.

*Ethical Approval Statement*. This study was conducted according to the guidelines of the Declaration of Helsinki and was approved by the Institutional Review Board of the University of the Ryukyus on 7 April 2015 (#936). All patients gave informed consent prior to her inclusion in the study.

## 2. Case Presentation

**Figure 1:**
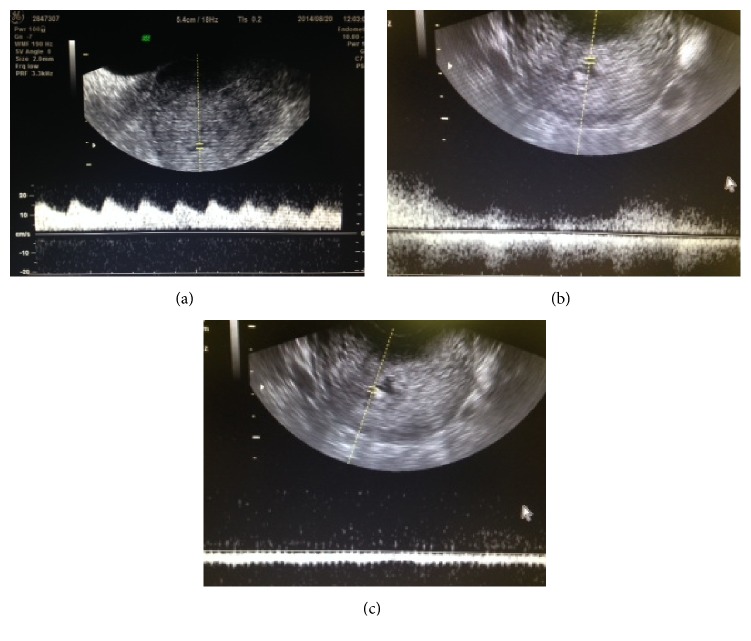
Diagnosis of AVM by Doppler examination (Patient 1). (a) Several arteries were observed in AVM. (b) A turbulent aspect in the fistula. (c) Venous flow in the intramural venous plexus.

**Figure 2:**
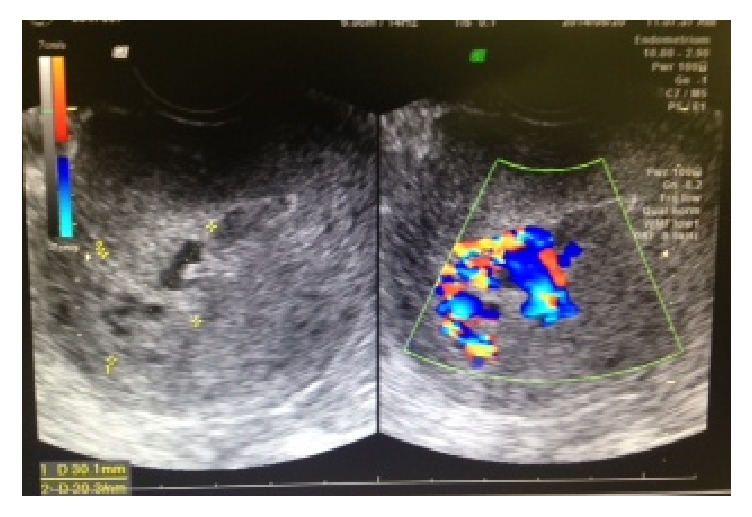
AVM depicted by ultrasonography using grayscale and color Doppler (Patient 1, mass measuring 22 × 19 mm in size).

**Figure 3:**
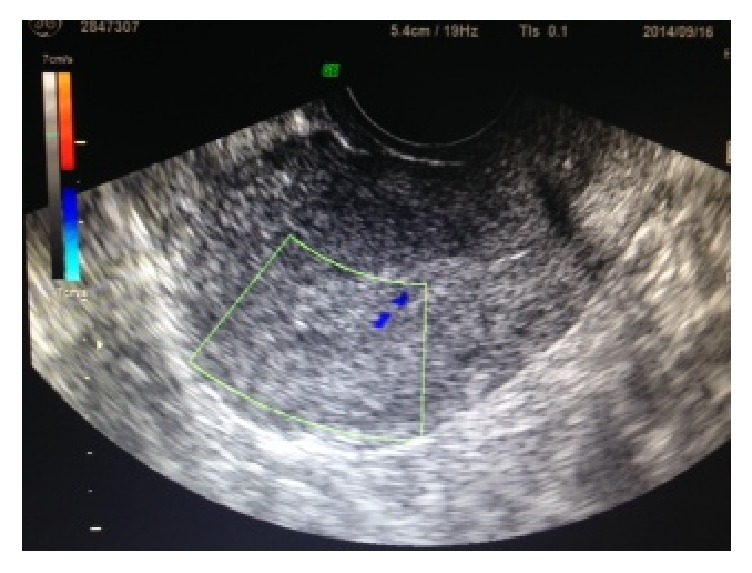
AVM spontaneously regressed after 6 weeks without UAE (Patient 1).

Clinical course of the three patients with AVM after miscarriage with conservative management are shown in Table 1. An AVM diagnosis was made using transvaginal ultrasound with grayscale and color and spectral Doppler imaging using the Voluson 3D power Doppler (GE Healthcare, Tokyo, Japan). Each examination comprised a standard grayscale ultrasound of the uterus in both the longitudinal and transverse planes. The endometrial thickness and presence/absence of retained products were recorded. Masses with abundant blood flow in the myometrium were shown as a mosaic color pattern using Doppler imaging. A venous-type flow in the intramural venous plexus, a turbulent aspect in the fistula, and a high-velocity low-resistance flow in the artery branch were observed in patients with AVM (Figure 1). The size of AVM was measured using ultrasonography. Patient 1 received medroxyprogesterone acetate therapy for stage IA endometrial cancer and grade 1 endometrioid adenocarcinoma. Following remission, she underwent in vitro fertilization-embryo transfer (IVF-ET) and became pregnant by freeze-thawed embryo transfer (ET). However, the pregnancy was a blighted ovum, and D&C was performed. Ultrasonography performed 2 weeks later revealed a cyst with a low-level echo and abundant blood flow. A mass measuring 22 × 19 mm in size with abundant blood flow in the myometrium was shown on Doppler imaging as a mosaic color pattern (Figures 1 and 2). As no vaginal bleeding was observed, we opted for conservative management. At diagnosis, the patient's estradiol level was 530 pg/mL, and a functional cyst was observed in the right ovary. Considering that the elevated estradiol level could have affected the growing AVM, a gonadotropin-releasing hormone (GnRH) agonist (900 *μ*g/day Suprecur®; Mochida Pharmaceutical Co., Ltd., Tokyo, Japan) was administered for 4 weeks. Furthermore, methylergometrine maleate was administered for 3 weeks to promote uterine contraction. Four weeks following diagnosis, the patient's estradiol level dropped to 5 pg/mL, the hCG level dropped to 4.9 mIU/mL, and the AVM disappeared (Figure 3). Five months after the disappearance of AVM, hysteroscopy revealed no abnormal findings in the uterus. The patient became pregnant by freeze-thawed ET and delivered a healthy infant.

**Figure 4:**
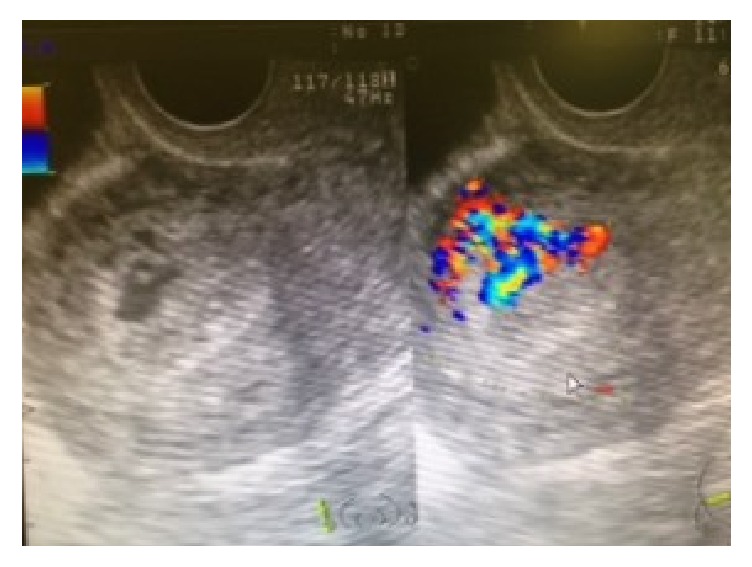
AVM depicted by ultrasonography using grayscale and color Doppler (Patient 2, mass measuring 16 × 6 mm in size).

Patient 2 also conceived by IVF-ET. However, it resulted in missed abortion, and she underwent D&C. She showed abnormal vessels (10 × 3 mm) in the myometrium on ultrasonography 1 day after D&C. After 1 week, the abnormal vessels had grown to a size of 16 × 6 mm with abundant blood flow, which was shown on Doppler as a mosaic color pattern (Figure 4). No vaginal bleeding was observed. Because her estradiol level was normal (42 pg/mL), no GnRH agonist was administered. Methylergometrine maleate was administered for 3 weeks to promote uterine contraction. Four weeks after D&C, AVM disappeared. The patient is currently undergoing IVF treatment.

**Figure 5:**
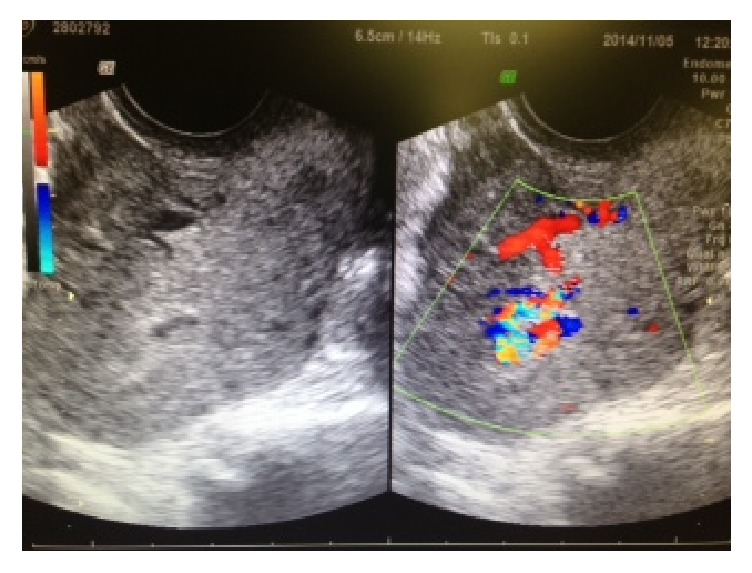
AVM depicted by ultrasonography using grayscale and color Doppler (Patient 3, mass measuring 20 × 15 mm in size).

Patient 3 had become pregnant by IVF-ET but had a missed abortion at 8 weeks of gestation, which ended in a complete abortion without D&C. Ultrasonography performed 1 week later revealed a cyst with a low-level echo (6 × 2 mm) and blood flow in the myometrium. Methylergometrine maleate was administered for 5 days. After 3 weeks, the cyst had grown to 20 × 15 mm with abundant blood flow (Figure 5). No vaginal bleeding was observed. Because the patient's estradiol level was 2,552 pg/mL and multiple functional cysts were observed in the right ovary, a GnRH agonist was administered. Six weeks after complete abortion, AVM disappeared and the patient's estradiol level dropped to 30.2 pg/mL. The patient is currently undergoing IVF treatment. Her hCG level was 294.1 mIU/mL on the day of AVM diagnosis, but it spontaneously decreased to 14.3 mIU/mL.

## 3. Discussion

Data regarding AVM following miscarriage are limited. Although the number of cases is small, we presented detailed cases of conservative management. Methylergometrine maleate was administered to all three cases to promote uterine contraction. A GnRH agonist was administered to cases 1 and 2 in whom estradiol levels were >300 pg/mL when AVM was diagnosed, which could have affected the growing AVM. In three patients with AVM without vaginal bleeding, the mass spontaneously disappeared without performing UAE. AVM does not always cause massive vaginal bleeding and spontaneously disappears in some patients. Therefore, for women concerned about ovarian insufficiency, particularly those undergoing fertility treatments, performing UAE should be carefully considered.

Although AVM-induced massive vaginal hemorrhage is considered a rare disease, it is possible that the actual incidence is higher because of cases not being diagnosed [1]. Asymptomatic AVM might spontaneously disappear without diagnosis. These three patients experienced miscarriage following IVF; therefore, we were able to diagnose AVM after performing frequent ultrasound examinations to determine when the next treatment should begin. Thus, in women undergoing fertility treatment, AVM may be diagnosed early, and it may be a mild form that spontaneously regresses. Conversely, women who are not infertile have fewer reasons to undergo ultrasound examination following a miscarriage. In these patients, AVM may be overlooked and may remain undiagnosed.

Blood flow in the myometrium, verified by color Doppler on day 3 and at 6 weeks following normal delivery, has been reported [16]. Of 93 cases following vaginal delivery, enhanced myometrial vascularity (EMV) with locally increased blood flow in the myometrium at the placental bed was observed in 50.5% on day 3 following delivery. However, EMV was observed in only 3.9% at 6 weeks, and it spontaneously disappeared in most cases. EMV is found in the myometrium at the site of placental attachment and is associated with retained placenta. However, no relationship has been observed between EMV and postpartum bleeding. If EMV is observed without vaginal bleeding, treatment is considered unnecessary [16]. On the basis of these reports, we can infer that a small amount of retained placenta following a normal delivery is related to the abnormal blood flow in the myometrium. AVM has also been suggested to be associated with retained placenta and villi [7]; therefore, it is difficult to distinguish between AVM and EMV that does not require treatment. Hence, we should consider expectant management for AVM without bleeding.

There are many reports of UAE for uterine myoma [17–19]. However, a simple comparison is impossible because metal coils are often used in these cases. Several reports on UAE, used to treat AVM after miscarriage, have described effects on ovarian function and adverse effects on subsequent pregnancies [20]. Inoue reported findings from 211 women who underwent UAE for postpartum hemorrhage [20]. Of the 113 women who underwent postoperative follow-up, amenorrhea developed in seven, Asherman's syndrome in four, endometritis in seven, and uterine necrosis in three. Placenta accreta was observed in five of 30 women (16.7%) who conceived following UAE.

When conservative treatment is selected for AVM, the use of methylergometrine maleate [11, 13] and GnRH agonists [12, 13] has been reported. Methylergometrine maleate is used to reduce AVM blood flow by promoting uterine contraction rather than directly acting on AVM. Estrogen is believed to contribute to vascular endothelial differentiation, and the use of GnRH agonists inhibits estrogen and thus might inhibit AVM progression [13]. GnRH agonist was administered to women with elevated estrogen levels of >300 pg/mL. We made this the cut-off value because it is the average value for the ovulation phase in normal reproductive women. However, it is unclear whether AVM disappearance was because of these agents or if it spontaneously occurred. Other reports have described treatment with follow-up observation only and without the use of drugs [1, 3, 14]. Timmerman prospectively performed expectant management in 30 patients with AVM who were diagnosed by ultrasonography and color Doppler [15]. Eight women required UAE for massive vaginal bleeding; however, in 22 women (73%), AVM spontaneously disappeared. Lee prospectively analyzed 75 patients with AVM and chose to perform UAE only in women who were anemic or hemodynamically unstable because of vaginal bleeding [3]. In 45 women (60%), AVM spontaneously disappeared without UAE. Evaluation by ultrasound and color Doppler showed the strongest association between low peak systolic velocity (cm/s) and the ability to undergo expectant management. Our department did not assess patient blood flow, which is a limitation that we plan to examine in the future. Furthermore, it is difficult to select a cut-off value for conservative management, but masses measuring < 3 cm without genital bleeding should be considered for conservative management.

In cases of AVM for which UAE is performed, early recurrence can develop with choriocarcinoma diagnosed after a subsequent hysterectomy [21]. Therefore, when considering uterine preservation, the possibility of trophoblastic disease should be considered. We observed no abnormal elevation of hCG levels in the three patients; therefore, trophoblastic disease was ruled out.

In conclusion, to avoid ovarian insufficiency caused by UAE and potential complications in subsequent pregnancies, we believe that conservative management should be considered as the primary approach in hemodynamically stable patients, particularly in those undergoing fertility treatment.

## Figures and Tables

**Table 1:** Clinical course of the three patients with AVM after miscarriage with conservative management.

	Age (y)	Conception	Surgery after miscarriage	Size of AVM (mm)	Hemorrhage	Estradiol level at AVM diagnosis (pg/mL)	hCG level at AVM diagnosis (mIU/mL)	Treatment	Time of spontaneous regression	Pregnancy after regressive AVM
1	37	IVF	D&C	22 × 19	(—)	530.0	93.4	Methylergometrine maleate for 3 weeks + GnRHa for 4 weeks	6 weeks after D&C	Term delivery
2	41	IVF	D&C	16 × 6	(—)	42	32.3	Methylergometrine maleate for 3 weeks	4 weeks after D&C	Under IVF treatment
3	41	IVF	Spontaneous abortion	20 × 15	(—)	2,552	294.1	Methylergometrine maleate for 5 days + GnRHa for 3 weeks	6 weeks after miscarriage	Under IVF treatment
